# Implantation of an extravascular defibrillator with a preexisting endocardial dual-chamber defibrillator: When 2 defibrillators share 1 heart

**DOI:** 10.1016/j.hrcr.2025.12.014

**Published:** 2026-01-08

**Authors:** Mathieu Echivard, Hugo Pegorer-Sfes, Luc Freysz, Arnaud Olivier

**Affiliations:** Department of Cardiology, CHRU Nancy, Vandoeuvre-les-Nancy, France

**Keywords:** Implantable cardioverter-defibrillator, Extravascular ICD, Endocardial pacing, Lead-related complication, Device–device interaction, Defibrillation

**Key Teaching Points**

• The combination of an extravascular implantable cardioverter-defibrillator (EV-ICD) and an endocardial dual-chamber system (including an implantable cardioverter-defibrillator) seems feasible and may represent a valuable option for patients requiring both pacing and defibrillation, particularly in the presence of complex venous anatomy or high procedural risk.
• Individualized patient assessment is essential, taking into account venous anatomy, lead position, pacing needs, and arrhythmic risk when considering such dual-device configurations.
• Systematic testing for device–device interaction is mandatory, including high-output pacing and arrhythmia induction, to ensure appropriate sensing and absence of interference. This case also outlines manufacturer considerations to optimize programming and safety.
• Given that endocardial pacing and EV-ICD may increasingly coexist, further clinical experience and long-term follow-up are required to confirm the safety and reliability of this dual-device approach.

## Introduction

Implantable cardioverter-defibrillators (ICDs) are a cornerstone therapy for the prevention of life-threatening ventricular arrhythmias.^1^ However, transvenous ICD systems carry long-term risks such as infection, lead failure, and venous thrombosis—particularly concerning in younger patients who may require lifelong device therapy.

To address these limitations, nontransvenous defibrillation systems have been developed. The subcutaneous ICD eliminates the need for transvenous leads but does not provide pacing capabilities.^2^ More recently, the extravascular ICD (EV-ICD) has been introduced.^3^^,^^4^ By placing the defibrillation lead in a substernal position, the EV-ICD maintains effective defibrillation thresholds while also enabling antitachycardia pacing through a single device.

Despite these advantages, EV-ICD implantation is currently contraindicated in patients with existing dual-chamber pacing systems because of the potential for device–device interaction that could impair sensing or therapy delivery. Such a configuration has not been formally evaluated, and its safety and functionality remain uncertain.

We report here the first case of an EV-ICD implanted in a patient already equipped with a dual-chamber ICD, highlighting the feasibility and practical considerations of this novel, off-label approach.

## Case report

A 71-year-old man with ischemic cardiomyopathy, a long history of ventricular tachycardia (VT), and multiple ICD system revisions was referred for evaluation after an inappropriate ICD shock.

He had experienced a myocardial infarction approximately 25 years earlier, resulting in severe left ventricular dysfunction (left ventricular ejection fraction 25%). A single-chamber ICD was implanted a few years later after syncope and inducible VT during programmed ventricular stimulation. Over the next 2 decades, the ICD system required multiple interventions owing to lead-related complications and generator replacements. The first generator replacement occurred roughly midway through this period. Several years later, a right ventricular (RV) lead fracture necessitated the implantation of an additional lead after a failed extraction attempt, with generator replacement performed concurrently. A few years afterward, the system was upgraded to a dual-chamber ICD to manage concomitant symptomatic sinus node dysfunction. The patient had experienced multiple sustained VT episodes approximately 10 years prior to presentation, with a progressively increasing burden over 3 consecutive years. He subsequently underwent radiofrequency ablation, after which only 1 recurrence occurred a couple of years later—a fast VT episode (250 beats per minute) successfully terminated by an ICD shock.

The patient presented after experiencing a single inappropriate ICD shock. Device interrogation revealed noise on the RV lead preceding the shock. This noise was misinterpreted as ventricular fibrillation (VF), leading to inappropriate therapy delivery during sinus rhythm. All other parameters—including shock impedance—remained stable. The episode was considered consistent with a new lead fracture, specifically involving the RV pace–sense conductor. In summary, the patient presented with 1 functional atrial lead (SOLIA, Biotronik) connected to a recently implanted device (ACTICOR 7 DR-T, Biotronik; estimated battery longevity close to 10 years), 1 abandoned RV lead (ELA Medical), and 1 fractured RV lead (RELIANCE 4-FRONT, Boston Scientific) remaining in situ on the left side. This occurred in the context of ischemic cardiomyopathy with a history of VT and sinus node dysfunction, requiring atrial pacing and the capability for defibrillation therapy. The left pectoral pocket had housed 4 previous ICD generators, reflecting a substantial cumulative burden from previous revisions and hardware.

4 management strategies were evaluated:
(1) Adding a new RV lead, which was limited by venous access and increased hardware burden
(2) Implanting a new right-sided ICD system, also resulting in excessive hardware and infection risk
(3) Extracting existing leads, a high-risk procedure given their number and chronicity
(4) Implanting a subcutaneous ICD alongside the existing system, excluded because of failed preimplant screening.

Evaluation of an EV-ICD was undertaken; however, this option was formally contraindicated because of the presence of a dual-chamber pacing system and the potential for device–device interaction that could impair sensing or therapy delivery.

**Figure 1:**
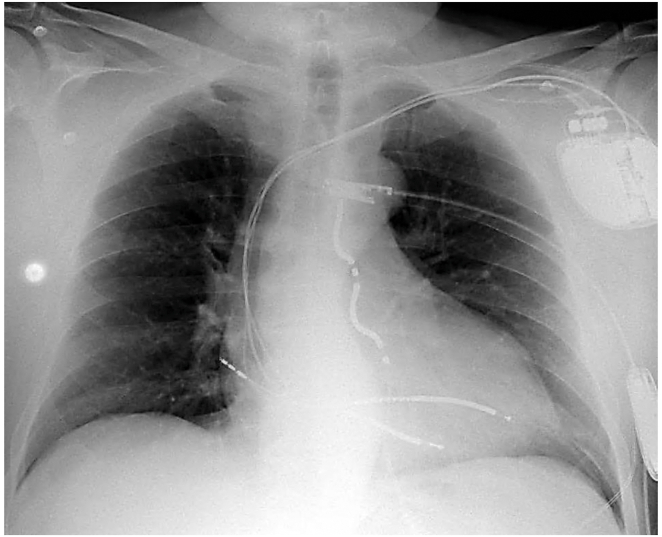
Postoperative chest radiograph showing a dual-chamber endocardial implantable cardioverter-defibrillator, an abandoned right ventricular lead, and an extravascular implantable cardioverter-defibrillator system.

In light of the lack of viable transvenous options, the high procedural risk of lead extraction, and the patient’s informed consent, an off-label EV-ICD implantation was ultimately performed. The postoperative chest radiograph is presented in Figure 1. The preexisting atrial lead was positioned on the lateral atrial wall. The Epsila EV-ICD lead was tunneled twice: the first, more lateral trajectory was unsuccessful, whereas the second, more axial trajectory was successful.

**Figure 2:**
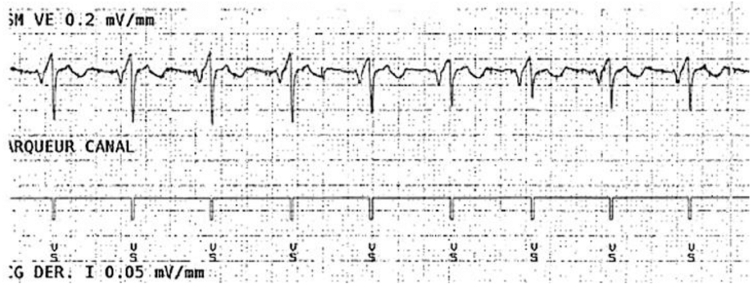
Electrogram of the extravascular implantable cardioverter-defibrillator showing atrial pacing at 6 V and 0.4 ms in AAI mode with the extravascular implantable cardioverter-defibrillator sensing programmed at 0.15 mV. No oversensing or double counting was observed during high-output atrial pacing, confirming the absence of device–device interaction.

**Figure 3:**
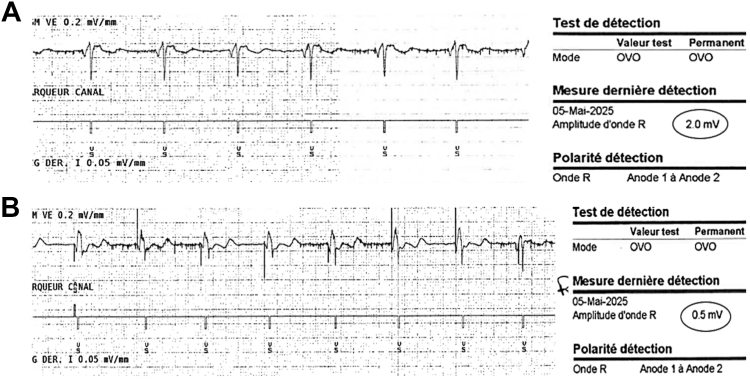
Extravascular implantable cardioverter-defibrillator sensing during spontaneous and paced ventricular rhythms. **A:** During spontaneous rhythm, the sensed R-wave amplitude was 2.0 mV. **B:** During ventricular pacing at 3.5 V and 0.4 ms in DDD mode, no pacing artifact oversensing occurred, and the sensed R-wave amplitude decreased to 0.5 mV, confirming the absence of interference between systems.

To assess potential device–device interactions, EV-ICD sensing was evaluated during high-output atrial and ventricular pacing, with the EV-ICD sensitivity set at 0.15 mV. During bipolar atrial pacing at 6 V and 0.4 ms in AAI mode, no oversensing or double counting was observed (Figure 2). During spontaneous ventricular rhythm, the sensed R-wave amplitude was 2 mV. With bipolar ventricular pacing at 3.5 V and 0.4 ms in DDD mode, no pacing artifact oversensing occurred, although the sensed R-wave amplitude decreased to 0.5 mV (Figure 3).

Defibrillator testing was performed at the end of the procedure. VF was successfully induced by 50-Hz stimulation and appropriately detected, but the initial 30-J shock failed to terminate the arrhythmia. A second test demonstrated appropriate detection of induced VF and effective shock delivery at 40 J, with a shock impedance of 90 Ω.

Sensitivity settings of the endocardial leads were left unchanged to maintain high sensitivity for the detection of low-voltage arrhythmias such as VF. No pacing was observed during VF episodes.

The preexisting endocardial ICD system was retained to provide pacing—primarily atrial for chronotropic insufficiency and ventricular when necessary. Given that no atrioventricular block had ever been documented, RV lead replacement solely for pacing was not considered. Given the infrequency of RV noise, adequate sensing and pacing thresholds, and the absence of device–device interaction in DDD mode, RV pacing capability was maintained. Preserving this functionality offered a potential—although unlikely—safeguard against high-degree conduction disturbances, particularly in light of a low yet meaningful ventricular pacing burden (≈10%) attributed to first-degree atrioventricular block. Because the patient was not atrial pacing dependent, transient RV noise–related atrial pacing inhibition was not considered clinically relevant. Daily remote monitoring ensured continuous assessment of RV lead parameters during follow-up.

The device was programmed in ADIR-DDDR mode (55–120 beats per minute), with all defibrillation therapies disabled. The EV-ICD sensitivity was programmed at 0.15 mV (nominal setting). Postshock and pause-prevention pacing functions were deactivated to avoid competitive pacing, and automatic Wavelet collection was disabled (the final programming of both devices is presented in Table 1).

**Table 1:** Final programming of the endocardial ICD and EV-ICD

Parameter	Endocardial ICD	EV-ICD
Primary function	Pacing system preserved (mainly atrial) in the context of sinus node dysfunction	Ventricular arrhythmia detection and defibrillation
Sensing	Atrial sensitivity: 0.6 mV Ventricular sensitivity: 0.8 mV	Sensitivity: 0.15 mV (nominal)
Pacing mode	ADIR-DDDR, 55–120 bpm	—
Pacing output	Atrial: 1.5 V / 0.40 ms (bipolar) Ventricular: 2.0 V / 0.40 ms (bipolar)	—
Postshock and pause-prevention pacing	—	Disabled
Defibrillation therapies	Disabled	Enabled
VT detection zone	—	182–231 bpm
VT therapy	—	ATP × 3 bursts, then 5 shocks at 40 J
VF detection zone	—	>231 bpm
VF therapy	—	6 shocks at 40 J
Discrimination	—	All discrimination algorithms enabled; Wavelet automatic collection disabled

ATP = antitachycardia pacing; bpm = beats per minute; EV-ICD = extravascular implantable cardioverter-defibrillator; ICD = implantable cardioverter-defibrillator; VF = ventricular fibrillation; VT = ventricular tachycardia.

The patient recovered uneventfully and was discharged 24 hours after the procedure. After a 6-month follow-up, both pacing and defibrillation functions remained stable, with no evidence of inappropriate sensing, oversensing, or device interference, despite 82% atrial pacing and 20% ventricular pacing (likely related to prolonged atrioventricular delay). No ventricular arrhythmias occurred during follow-up.

## Discussion

This case illustrates a complex scenario of ICD management in a patient requiring both defibrillation and pacing support. The EV-ICD provided an effective solution, avoiding the procedural and long-term risks associated with additional transvenous leads.

No oversensing or double counting was observed during pacing from either lead under both normal conditions (including a 6-month follow-up) and worst-case scenarios, such as high-output pacing or arrhythmia. Although a decrease in sensed R-wave amplitude was noted during ventricular pacing, this finding was not clinically relevant, given that the purpose of sensing is to detect spontaneous ventricular arrhythmias. Interestingly, this discrepancy resolved at the 3-month follow-up, with R-wave amplitudes during spontaneous and paced ventricular rhythms becoming similar, ranging from 1.5 to 3 mV depending on the patient’s position, which is well above the ≥1 mV threshold typically expected for EV-ICDs.^4^

The 30-J shock failure was the only issue encountered during the procedure. Although anesthetic agents may influence defibrillation efficacy, the patient was not receiving any antiarrhythmic therapy known to increase the defibrillation threshold. It is conceivable that the presence of the 2 coils contributed to this initial failure. However, had the subsequent 40-J shock also failed, repeat defibrillation testing would have been performed later, given that air entrapment during the procedure can occasionally cause transient defibrillation failure.

Few cases of combined leadless pacemaker and EV-ICD implantation have been reported recently.^5^^,^^6^ However, to the best of our knowledge, only 2 cases combining an EV-ICD and a dual-chamber transvenous pacemaker have been reported to date—one involving pacemaker implantation in a patient with a preexisting EV-ICD^7^ and the other describing EV-ICD implantation in a patient with a preexisting pacemaker.^8^ In those reports, simultaneous implantation seemed safe and feasible, with no interference between the 2 devices.

Our case seems to be the first to describe the coexistence of an EV-ICD and a dual-chamber ICD system. The presence of ICD coils, in particular, could have raised concerns regarding defibrillation efficacy; however, despite an initial 30-J shock failure, defibrillation ultimately performed adequately, even in the presence of 2 coils. It demonstrates that combining both systems is feasible, provided that careful case-by-case assessment is performed. Individual patient characteristics—including venous anatomy, atrial lead position, pacing requirements, and arrhythmic risk—must be considered. In our case, the atrial lead position on the lateral wall rather than in the appendage may have been a favorable factor.

In cases of off-label EV-ICD implantation combined with an existing pacing system, specific programming considerations are essential to ensure the safe coexistence of both devices. According to Medtronic’s considerations for such configurations, endocardial device settings should be optimized by tailoring the output amplitude to the lowest level that reliably achieves capture, using bipolar pacing mode, and maintaining high ventricular sensitivity to avoid undersensing of VF or antitachycardia pacing delivered by the EV-ICD.

For the EV-ICD, sensitivity should be programmed to ≥0.15 mV to minimize the risk of oversensing pacing artifacts. Postshock and pause-prevention pacing should be deactivated to prevent competitive pacing, and automatic Wavelet collection should be turned off. Manual Wavelet template acquisition should be performed during intrinsic rhythm to ensure accurate morphology-based sensing.

If defibrillation threshold testing is performed, the endocardial device should first be programmed according to these parameters. VF can then be induced to confirm appropriate detection and effective shock delivery by the EV-ICD, followed by verification of the proper function of both systems after therapy.

More cases of simultaneous EV-ICD use and dual-chamber pacing are needed to further assess safety, given that endocardial pacing and EV-ICDs are likely to increasingly coexist—either in EV-ICD patients who later require atrial or ventricular pacing or in previously paced patients who subsequently require defibrillation therapy.

## Conclusion

This case illustrates the feasibility of combining endocardial dual-chamber pacing with an EV-ICD system and demonstrates that the coexistence of an endocardial ICD and an EV-ICD is possible without adverse interactions. Such an approach requires careful, individualized assessment based on the patient’s anatomy, lead configuration, pacing needs, and arrhythmic profile.

Systematic evaluation of device–device interaction is essential, along with adherence to manufacturer-specific programming recommendations to ensure safe and reliable function of both systems.

Further cases are needed to confirm the long-term safety of this strategy, given that endocardial pacing and EV-ICDs are likely to increasingly coexist, with patients potentially requiring pacing or defibrillation at different stages of their disease.

## Disclosures

Mathieu Echivard reports receiving honoraria from Boehringer Ingelheim, Biotronik, Novartis, Zoll, and Medtronic. Arnaud Olivier received travel support from Servier, Novartis, Novo Nordisk, MicroPort, and AstraZeneca; served on the advisory board for MicroPort and AstraZeneca; and received consulting fees, honoraria, and travel support from Medtronic. All other authors report no conflicts of interest.
